# Traité des Nerfs et de leurs Maladies

**Published:** 1781-01

**Authors:** 


*et de la Société Royale de Medecine de Paris.* 8vo. Lausanne. 6 vols.

IT is well known that nervous diseases are much more frequent now than they were formerly. Our manner of living, the luxury that prevails in large cities, and extends to persons of every class, the immoderate use of tea, coffee, wine, and spirituous liquors, all concur in disposing us to complaints of this sort. The small number of nervous disorders that existed in ancient times, is, doubtless, one of the reasons why the earlier medical writers have left us but little on the subject; for, excepting palsy and spasm, there were no other affections which they considered as nervous; and yet in perusing their observations, we may easily distinguish cases, to which indeed they have given no appellation, but which evidently appear to have been what would now-a-days be considered as disorders of the nerves. Another reason likewise, in the opinion of the author of the work before us, why the ancient physicians omitted to ascertain the true cause of these diseases, is the difficulty with which they are distinguished. A long series of observations was necessary for this purpose, and a more perfect knowledge of phy-

ſiology than the ancients could poſſibly poſſeſs. The celebrated Sydenham is almoſt univerſally conſidered as the firſt medical writer who clearly aſcertained the variety of ſymptoms occaſioned by diſeaſes of this claſs, and who remarked at the ſame time, that all their ſymptoms depend upon too much or too little nervous energy. But M. Tiſſot obſerves, in his Preface, that Sydenham was not the firſt who referred hyſteric affection to the nerves; this diſcovery having been made thirty years before his time, by Charles Piſo, a phyſician of Lorraine, in a work entitled, *De Morbis ex colluvie et diluvie ortis.* Piſo's book, which abounds with the phyſiological errors of the preceding ages, was publiſhed in 1618, ten years before the appearance of Harvey's great work, which made ſo thorough a change in phyſiological reaſoning.

Our author remarks, that after the time of Sydenham and Willis, both of whom made many valuable obſervations on affections of the nerves, this claſs of diſeaſes was ſo far from being improved, that ſucceeding writers returned to the old and erroneous doctrines, ſeemingly through ignorance of what had been done by their predeceſſors; ſo that in 1750, the works works of Cheyne and Hoffmann were almoſt the only valuable ones on this ſubject; and yet neither of theſe contains much more than what is to be found in the writings of the two juſt now mentioned. Since that time, the works of Boerhaave, Whytt, and Lorry, on this ſubject, have made their appearance. Each of theſe contains many excellent things, but they are all of them very different in their plan from the preſent work, on which the author has employed himſelf it ſeems, at times, ever ſince the year 1759. The third volume, which treats on the epilepſy, having been the firſt finiſhed, was publiſhed in 1770. Ill health, and a variety of avocations, have retarded the appearance of the remainder till now.

M. Tiſſot begins by deſcribing firſt the anatomy, and then the phyſiology of the nerves; theſe two parts fill the whole of the firſt part of the firſt volume. "I am fearful (ſays he) that many phy-"ſicians will think it too long, and be of opinion, "that a very bulky volume of anatomy and phy-"ſiology is miſplaced at the head of a practical "work; but I am ſo thoroughly convinced, "that it is impoſſible to form an exact idea of "a diſeaſe without knowing both the parts in

" which it is feated, and their functions in
" a healthy ftate, that I dare affirm, that the
" fmall progrefs made in practice, is owing to
" the little fkill phyficians have in anatomy and
" phyfiology. I have obferved, that in pro-
" portion as we improve ourfelves in thefe two
" branches of fcience, we acquire a greater rea-
" dinefs at diftinguifhing the caufes of difeafes,
" and of courfe their true indications."

He then proceeds to point out the plan of his work, which confifts in proceeding from the phyfiology, to the pathology and cure of nervous difeafes.

After extending his anatomical defcriptions through 190 pages, and tracing the nerves from their origin to the parts in which they are dif-tributed, our author gives a fhort recapitulation of what he has advanced, by re-afcending, as it were, from thefe parts to the nerves, and pointing out in a general way thofe which are diftri-buted to each. The remainder of the firft part of the firft volume is employed on the phy-fiology of the nerves. In each of thefe parts we meet with a judicious compilation of what has been written by others on thefe fubjects, but without the addition of any thing new. He relates relates the different fyftems that have prevailed with regard to the functions of the nervous fyftem, and agrees with the generality of modern writers, in fuppofing the exiftence of a nervous fluid, but he rejects the notion adopted by M. Lieutaud, that there are two kinds of animal fpirits, the one intended for motion, and the other for fenfation. Speculating with regard to the nature of this fuppofed fluid, and the quicknefs of its motion in mufcular action, he is led to notice its different rapidity in different animals, and obferves that this rapidity is probably greateft in an infect defcribed by M. de l'Ifle, in the *Hift. de l'Academie des Sciences* for 1711. This infect it feems, which is fo minute as to be almoft invifible, makes 1080 fteps in a fecond, without advancing more than fix inches in that time.

The fecond part of the firft volume (or rather what we would call the fecond volume, for it is printed and paged feparately from the firft) begins with obfervations on the effects of poifons, which M. Tiffot contends are by no means foreign to his work, not only becaufe they include all the fymptoms of nervous diforders, but becaufe they are or may be reckoned amongft amongſt the cauſes of theſe diſeaſes, perſons who have had the misfortune to be poiſoned, being almoſt always ſubject to them during the remainder of their lives; and accordingly, adds our author, Boerhaave, in his treatiſe on diſorders of the nerves, employs almoſt ſixty pages on poiſons.

Our author ſets out with remarking, that if we except a ſmall number that appear to act by their ſtypticity, all poiſons exert their effects by means of a very acrid and irritating principle, that is fixed in ſome, and extremely volatile in others. He obſerves, that poiſons have been diſtinguiſhed into mineral, vegetable, and animal, but that this arrangement, which was firſt introduced by Mead, throws no light on their action. Our author confines himſelf to the effect of the irritation, which may be applied; 1ſt, by reſpiration to the different parts of the pituitary membrane. 2dly, By the cutaneous inhalation. 3dly, By wounds. 4thly, By deglutition. 5thly, By clyſters. 6thly, By injections, either into the blood veſſels, or cavities of the thorax and abdomen, or into the hollow viſcera. To the firſt of theſe claſſes, he obſerves, may be referred all thoſe extremely volatile volatile poiſons which kill the moment they are reſpired, and likewiſe all thoſe leſs powerful ones which without killing prove extremely noxious. Certain flowers and chymical exhalations, mephitic vapours, the inflammable air of ſtagnant waters, vapours ariſing from animal and vegetable ſubſtances in a ſtate of putrefaction, &c. are of this kind; and although ſeveral of theſe prove deſtructive, chiefly by vitiating the air, and infecting the fluids, yet it has been very clearly perceived, that another cauſe of death, which exiſts perhaps in all of them, and in ſeveral is the principal agent, is the exceſſive acrimony which excites a violent ſpaſm of the bronchiæ that proves ſuddenly fatal. Our author quotes from the Philoſophical Tranſactions the caſe of a woman who was killed by ſleeping in a chamber in which there was a great quantity of roſes. He mentions another inſtance on the authority of Triller, of a ſimilar effect produced by violets. He adds, that there are ſome plants which have but little odour, aud yet their odoriferous part, ſmall as it is, is truly poiſonous. He obſerves that Boerhaave, after informing us that at one time he had like to have been killed by imprudently venturing venturing too near the fumes of ſome ſpirit of vitriol he was preparing according to Van Helmont's proceſs, and that at another he was expoſed to ſimilar danger from the vapour of putrid urine, relates two facts which are ſtill more curious. One of theſe concerns Tachenius, who on opening a veſſel in which he had been ſubliming arſenic, was inſtantly ſeized with excruciating pain at the pit of the ſtomach, ſyncope, cold ſweat, vomiting, and a general ſpaſm, which gave way only by degrees to a vegetable diet; the other is of a painter, who upon opening a box in which he had long kept ſome realgar, perceived a ſlight ſmell, and immediately fell into a ſyncope, from which he was with difficulty recovered. It is very evident, remarks M. Tiſſot, that both theſe accidents were occaſioned wholly by the irritation of the nervous ſyſtem. Fernelius, ſays he, ſpeaks of a ſilverſmith, who by uſing mercury in gilding ſuddenly became deaf and dumb, and loſt his underſtanding; and Etmuller having been expoſed by the breaking of a retort, to the fumes of ſulphur and antimony, found his breaſt ſo much irritated, that he continued to cough at times for upwards of a month. "Even the

"vapour

" vapour of simple sea water, adds our author,

" in a corrupted state, becomes one of the most

" active poisons. An instance of this sort is

" related in the Memoirs of the Acad. of Sc. for

" 1745, in which we are told, that a sailor fell

" down dead upon drawing up a bucket of sea

" water from the hold of a ship at Rochefort; six

" of his comrades, who were at some little distance

" from him, fell down deprived of their senses,

" and were seized with violent convulsions. The

" surgeon of the ship running to their assistance,

" experienced the same effects: the sailor who

" died voided blood from his mouth, nose, and

" ears; and his body was soon in so putrid a

" state, that it was impossible to open it. There

" are but too many instances of persons poi-

" soned by letters, by gloves, in a word, by

" breathing the slightest particle of a very vola-

" tile and oftentimes inodorous poison, for acri-

" mony may be of a nature capable of exciting

" a violent spasm, without affording any smell.

" The Emperor Henry VI. was poisoned by a

" pair of gloves; John King of Castile, accord-

" ing to some historians, by a pair of boots

" prepared by a Turk; and Lewis XIV. fear-

" ing there was a project for poisoning Philip V.

 " cautioned

" cautioned him not to open any letters, or
" make ufe of gloves, or fmell to any thing.
" There are not wanting inftances of poifoned
" candles; and fome hiftorians are of opinion,
" that the fecond Cardinal de Guife was poifoned
" in a proceffion at Avignon, by the fumes of
" the flambeaux that were carried before him.

" Boerhaave afferts, and no perfon was ever
" more deferving of being credited, that he
" knows of poifons capable of killing in the
" twinkling of an eye, without producing any
" fymptoms of difeafe. The exhalations of
" the manzanilla, a poifonous tree of America,
" that cannot be approached till care has been
" taken to diffipate its vapour: thofe of the
" Ahouai of the Weft Indies, and of the Ahouai
" of Madagafcar; thofe of the root of Bejuga
" when boiled, are dangerous only from their
" acrimony, and it is not by their rendering the
" air unfit for refpiration, that we can fufpect
" them of proving noxious, fince it is clearly
" demonftrated that vegetables reftore this qua-
" lity to air, when it has been deprived of it by
" other caufes; and that befides, the fymptoms
" they occafion, are not fuch as depend on that
" caufe."

Amongft

Among the poifons of this kind our author enumerates the exhalation that infects the *Samyel* or *Sanum*, a wind that blows in certain parts of Arabia, and proves almoft inftantaneoufly deftructive, corrupting the dead bodies with fo much rapidity, that the limbs may eafily be feparated from the trunk; and likewife the *Harmatan*, that blows in the month of January on the coaft of Guinea, and fometimes occafions blindnefs, and other baneful effects.

Peftilential poifons, fuch as the poifon of the plague, and the effluvia of putrid animal fubftances, in the opinion of M. Tiffot, act by producing palfy rather than fpafm.

Under this head of poifons that act upon the pituitary membrane, our author claffes fternutatories, and mentions a cafe that occurred to Boerhaave, of a man, who, by fnuffing up white hellebore that had been imprudently given to him, fell into fuch violent fneezings, that he would perhaps have died of Tetanos if the irritation had not been fpeedily removed.

Under the fecond head, that of abforption by the fkin, our author mentions Profeffor Monro's experiments defcribed in the Phyf. and Lit. Effays, and likewife a curious fact from Diemer-

 broeck, broeck, concerning an adventurer, who, during the plague of Nimeguen, diftributed at a very high price a kind of amulet, that was fufpended round the neck by way of prefervative. This amulet, which proved to be a preparation of arfenic, not only produced black puftules on the fkin, but likewife occafioned pains in the breaft, which ceafed upon its being removed. Another cafe is related from Wepfer, of a young woman at Bafil, who by rubbing her head with an ointment compofed of butter and arfenic, in order to deftroy vermin, brought on at firft violent pains, and afterwards a fwelling of her whole head, delirium, fever, and on the fixth day, death. A third inftance is quoted from Sproegel (*experim. circa venen*) of a woman at Gottingen, who having fprinkled fome powder of cobalt on her daughter's head as a cure for the tinea, had the mortification to fee her die in great agonies a few hours after. Dr. Sproegel covered fome ulcers in a dog with the fame powder, and the animal died in the fame manner. The fame phyfician afterwards fhaved the back of another dog, and after making fmall incifions in it, fprinkled them with about a drachm of arfenic; the animal immediately fell into violent convulconvulſions, accompanied with the ſtrongeſt efforts to vomit, and the ſame ſymptoms in ſhort as if it had taken the poiſon by the mouth; it died in convulſions at the end of five hours. The ſkin of the back was ſwelled and of a black livid colour; the ſtomach, inteſtines, pleura, breaſt, and lungs, were inflamed in the ſame manner as might have been expected if he had ſwallowed the poiſon. Boerhaave in his *Prælect.* ſect. 1122, relates a horrid inſtance of a ſimilar effect from a poiſonous ointment employed during the plague at Vienna. This ointment, which conſiſted of lard impregnated with the peſtilential virus, was uſed for the moſt villainous purpoſes, and when rubbed on the ſurface of the body proved ſpeedily fatal. M. Tiſſot remarks that he has often experienced alarming effects from the moſt ſimple ointments; there are ſome perſons, he obſerves, whoſe cutaneous nerves are ſo ſenſible, that ointments of every kind immediately, or at leaſt after a very ſhort time, produce an eryſipelatous eruption that he has ſeen ſpread over the whole body, and occaſion true nervous ſymptoms that have continued for ſeveral days; and among a variety of inſtances of very conſiderable irritation excited by the application of the bark of the *Daphne Laureola*

or or *Spurge Laurel*, which of late years has been ſo much in vogue, he remembers ſeveral in which he obſerved exactly the ſame ſymptoms as might have been expected from poiſon.

Our author remarks, that it is a well known fact that ſeveral poiſons, ſuch as the oil of tobacco, the poiſon of the viper, &c. that are extremely active when applied to a wound, occaſion no ill effect when ſwallowed. Thus we find that the Marſi and Pſyllii ſucked the wounds of the Roman ſoldiers the moment after they had been bit by the venomous ſerpents of Africa; and upon this ſame principle, Cozzi, viper-catcher to the grand duke of Tuſcany, ſwallowed a drachm of the poiſon of the viper, without being incommoded by it, although one or two drops of it were ſufficient to kill an animal when dropped into a wound.

M. Tiſſot obſerves, that different poiſons introduced into wounds, produce different effects; that of the aſp, for example, renders the patient lethargic; that of the *coluber ceraſtes* occaſions tetanos; that of the viper, jaundice; that of the ſeps, gangrene; while that of the *coluber dipſas* inflames the œſophagus, and excites an ardent thirſt; and that of the rattleſnake kills inſtantaneouſly, without producing any ſenſible effects. Our author remarks, that in animals that that have been killed by the bite of the viper no ſenſible change was to be diſcovered in the body, that could lead to a diſcovery of its mode of operation; but he preſumes it may be conſidered as a certainty that in almoſt all theſe caſes the nervous ſyſtem is ſingularly affected. He obſerves that Melchior Fries attributed too much to ſimple mechanical irritation, when he ſuppoſed that the viper diſcharged no poiſon into the wound, but proved noxious merely by irritating the part with its tooth; and that the late M. Pouteau, without denying the exiſtence of virus, was no leſs in the wrong, when he aſſerted that all the ſymptoms in ſuch caſes were occaſioned by local irritation, that is, by its effects on the wound.

After relating the effects of the bite of the rattleſnake, as given in the *Philoſ. Tranſ.* N° 399, our author quotes a curious fact from Tavernier concerning an Indian prince, whom he had occaſion to ſee put a criminal to death by running a poiſoned lance into one of his toes. Two ſurgeons, who were at hand, immediately amputated the toe, but this did not prevent the unhappy wretch from expiring very ſpeedily in convulſions. Bontius, adds M. Tiſſot, who practiſed practifed phyfic for a long time in the Eaft Indies, fays, that the moft ufual fymptom from wounds with poifoned arrows is a violent ecftafy; the patient feems intoxicated, ftaggers, and foon falls down dead; fymptoms that are evidently the effects of an injury done to the nervous fyftem.

As a ftriking inftance of the effects of poifons taken internally, our author quotes Wepfer's account of feven children poifoned by water hemlock, which they miftook for another plant.

Jacob Mæder, one of thefe children, aged fix years, returned home chearful, but in a fhort time complained of acute pain at the pit of his ftomach, and almoft in the fame inftant fell down fenfelefs, and made water with fo much force, that the flow of urine reached to the height of five feet; foon after he was attacked with violent convulfions; his jaws became fo clofed that it was impoffible to open them; he had an inceffant grinding of his teeth, his eyes were horridly diftorted, blood gufhed from his ears he had a violent hiccup, and frequent inclination to vomit; but his mouth was fo firmly fhut that it was impoffible for him to void any thing. At the epigaftrium a tumour appeared of of the fize of a man's fift, attended with confiderable pulfation; his arms and legs were affected by the ftrongeft convulfions, his head and trunk bent backwards formed an arch, under which another child was able to pafs. Thefe fymptoms relaxed for a moment, but foon returned with the fame violence, and he expired before this painful fcene had lafted half an hour.

Matthew Graff, a little after the death of Jacob Mæder, was attacked with vertigo, which at firft obliged him to fit down, but it was not long before he fell into violent convulfions, attended with opiftohotnos and locked jaw; they broke out fome of his teeth in order to get a little theriaca and vinegar into his ftomach, but his œfophagus was in fuch a ftate of fpafm, as would not allow him to fwallow; and a tumour that appeared at the pit of his ftomach, beat with fo much violence, that the preffure of the ftrongeft hand could not moderate its efforts. The paroxyfm continued in full force for half an hour, without any evacuation taking place, and the patient died.

The belly and face of Mæder had fwelled, but in Graff the fwelling was fo general, that it was impoſſible to get his cloaths off. From the moment of his death to that of his being interred, a great quantity of green froth came out of his mouth; and the ſame circumſtance occurred in Mæder.

The five others experienced ſimilar ſymptoms as the two that died; but having vomited up the poiſon by means of a ſolution of theriaca in vinegar, ſoon got well.

Boerhaave had occaſion to ſee eight children who were poiſoned by the ſame root, which produced the ſame ſymptoms as thoſe deſcribed by Wepfer. He gave to all of them a ſolution of white vitriol, and all who vomited recovered.

Our author obſerves, that ſome poiſons ſeem to act more upon certain nerves than others; the deadly nightſhade, for inſtance, affects the nerves of the eyes in a ſingular manner.

M. Tiſſot cloſes his remarks on the effects of poiſons with an account of the experiments made in this way on animals by Mortimer, Langriſh, Sproegel, and others.

In the following ſection our author attempts to explain the action of the nerves, and after offering his conjectures on this ſubject, proceeds to review what has been ſaid by different writers conconcerning their ganglions, and coverings, on both of which heads he ſeems chiefly to adopt the opinions of Haller. He next deſcribes the functions of the nerves, which he reduces to four, viz. 1. feeling; 2. determining muſcular action; 3. aſſiſting in nutrition; 4. aſſiſting the ſecretions.

[ *To be continued.* ]

