# Errata

**Published:** 1781-06

**Authors:** 


					-p. 33. 1
for 6 vols, read 3 vols.?p. 49,1. 14, for opifthatonos, read opifthotonos.

				

